# Advanced applications of cationic photosensitizer DHPC in photodynamic eradication of drug-resistant bacteria

**DOI:** 10.3389/fmicb.2026.1925192

**Published:** 2026-09-22

**Authors:** Hongshuang Qin, Lu Dong, Yinan Liu, Zitong Zhai, Xiaohui Jia, Delong Meng, Xueyi Sun, Yanxiang Guo, Li Feng, Tao Liu

**Affiliations:** 1 Department of Biological and Food Engineering, Lyuliang University, Lvliang, Shanxi, China; 2 School of Chemistry and Chemical Engineering, Shanxi University, Taiyuan, Shanxi, China; 3 Department of Chemistry and Chemical Engineering, Lyuliang University, Lvliang, Shanxi, China; 4 Department of Cardiology, The First Hospital of Shanxi Medical University, Taiyuan, Shanxi, China

**Keywords:** biofilm, cationic photosensitizer, drug-resistant bacteria, reactive oxygen species, wound healing

## Abstract

Photodynamic therapy (PDT) is increasingly viewed as a reliable and effective treatment for infections caused by antibiotic-resistant bacteria. However, the limited uptake of photosensitizers (PS) by bacteria remains a major challenge. Cationization enables PS to accumulate around bacterial cells, thereby promoting their intracellular delivery. In particular, cationic small-molecule PS are characterized by carrier-free bacterial enrichment, efficient biofilm penetration, high stability, straightforward synthesis, and moderate charges that minimize toxicity, making them a sustained focus in antimicrobial PS development. Herein, we investigate the potential of a cationic small-molecule PS, DHPC [(E)-8-(diethylamino)-2-(4-(diethylamino)-2-hydroxystyryl)-4-oxo-4H-pyrano[2,3-b]chromen-10-ium], for combating drug-resistant pathogens. Our results demonstrate that DHPC-mediated PDT (DHPC-PDT) effectively eradicates drug-resistant bacteria, including antibiotic-resistant *Staphylococcus aureus* and *Pseudomonas aeruginosa*, with greater efficacy compared to the commonly used photosensitizer methylene blue (MB). Furthermore, DHPC-PDT exhibits superior bactericidal activity compared to antitumor activity, with the two effects involving distinct underlying mechanisms, indicating that DHPC-PDT is better suited for antibacterial applications. It also shows a dual-action efficacy against biofilms, both inhibiting biofilm formation and eradicating mature biofilms. *In vivo*, DHPC-PDT reduces the bacterial burden in infected wounds, modulates the local inflammatory microenvironment, and accelerates wound healing, with preliminary evidence of favorable biocompatibility. This study presents DHPC as a potential PS for antimicrobial PDT.

## 1 Introduction

With the widespread and long-term use of antibiotics in clinical medicine, animal husbandry, and other fields, antimicrobial resistance (AMR) has become increasingly severe, with resistant strains continually emerging (GBD 2021 Antimicrobial Resistance Collaborators, 2024). Traditional antibiotics are often ineffective against drug-resistant infections, leading to prolonged hospitalization, elevated medical expenses, and increased mortality (Liu et al., 2026; Miller and Arias, 2024). To overcome this challenge in anti-infective treatment, new therapeutic strategies with a low risk of inducing resistance are urgently needed (Sati et al., 2025; Wang et al., 2025).

Photodynamic therapy (PDT) has been validated as a robust therapeutic modality for addressing antibiotic resistance (Triana et al., 2025). During PDT, photosensitizers (PS) absorb photons to generate reactive oxygen species (ROS), which oxidatively impair biomacromolecules and ultimately kill pathogenic bacteria (Antunes et al., 2021; Vatansever et al., 2013). Because of the rapid action and non-specific mechanism, PDT rarely induces drug resistance (Bispo et al., 2022; Piksa et al., 2023; Surur et al., 2024). However, most PS exhibit poor accumulation within bacteria, consequently compromising the photodynamic bactericidal efficacy (da Cunha et al., 2025; Shleeva et al., 2024; Zhou et al., 2025).

Cationization strategies enhance electrostatic interactions between PS and the negatively charged bacterial envelope, thereby promoting PS uptake and improving PDT efficacy (Chaves et al., 2024; Lin et al., 2018). Various cationic PS systems have been reported, including polymer-based, peptide-based, nanoparticle-based, and small-molecule platforms. Although these systems have contributed substantially to the field, they each suffer from inherent limitations. Excessive positive charges often lead to cytotoxicity, poor stability compromises their practical utility, and the relatively large size of polymers and nanoparticles restricts their penetration into biofilms and bacterial membranes (Bicker and Cobb, 2020; Ghosh et al., 2024). In view of these challenges, cationic small-molecule PS have remained a major focus in the development of novel antimicrobial agents. The intrinsic cationic groups allow small-molecule PS to accumulate around bacteria and facilitate their cellular uptake, without the need for auxiliary carriers or nano-assembly (Caruso et al., 2019; Digby et al., 2021). Their small size further allows efficient crossing of bacterial membranes and deep penetration into biofilms, while their moderate positive charges confer low toxicity and favorable biocompatibility. In addition, small-molecule PS exhibit excellent stability against degradation, along with straightforward and reproducible synthesis (Amorim et al., 2025). Taken together, these features make cationic small-molecule PS a highly attractive class of antimicrobial agents. However, the cationic small-molecule PS reported suffer from either short excitation wavelengths, oxygen dependence (which precludes activity under hypoxia), or poor bactericidal efficacy (Almodôvar et al., 2025; Maisch et al., 2014; Silva et al., 2024). Therefore, the development of novel cationic small-molecule PS with potent activity against drug-resistant pathogens is urgently required.

Our collaborators previously reported that cationic small-molecule PS DHPC [(E)-8-(diethylamino)-2-(4-(diethylamino)-2-hydroxystyryl)-4-oxo-4H-pyrano[2,3-b]chromen-10-ium] can generate ROS and kill tumor cells through PDT (Huang et al., 2025). However, DHPC-mediated PDT (DHPC-PDT) shows greater promise for antibacterial applications than for antitumor therapy, which is mainly attributed to the higher sensitivity of cationic PS toward bacteria (Mi et al., 2025; Savelyeva et al., 2023; Zhang et al., 2024). In addition, DHPC features a compact molecular scaffold, substituent-independent photodynamic activity, and abundant sites for chemical modification. Therefore, this study concentrates on the photodynamic antimicrobial activity of DHPC against drug-resistant bacteria. Our data reveal that DHPC-PDT effectively eradicates methicillin-resistant *Staphylococcus aureus* (MRSA) and multidrug-resistant *Pseudomonas aeruginosa* (*P. aeruginosa*). Notably, DHPC-PDT shows superior efficacy compared to methylene blue (MB) mediated PDT (MB-PDT) under identical experimental conditions. Moreover, the fully bactericidal condition of DHPC-PDT is inadequate for complete tumor cell elimination, confirming the advantage of DHPC-PDT in antibacterial applications. Additionally, DHPC-PDT is able to disrupt pre-formed biofilms, kill the embedded bacteria, and prevent biofilm formation. *In vivo* studies show that DHPC-PDT accelerates infected wound healing, alleviates wound inflammation, and exhibits favorable biocompatibility in preliminary experiments. This study provides a novel PS for PDT against drug-resistant bacteria.

## 2 Materials and methods

### 2.1 Bacterial strains and reagents

Methicillin-resistant *Staphylococcus aureus* (MRSA, SHMCC D10495, ATCC43300) and multidrug-resistant *Pseudomonas aeruginosa* (SHMCC D25207) were purchased from Shanghai Bioresource Collection Center (Shanghai, China). Hydroxyphenyl fluorescein (HPF, SJ-MD0109) was purchased from SparkJade (Shandong, China). Fluorescein diacetate (FDA, F8040), crystal violet (C8470), ATP Content Assay Kit (BC0305) and Superoxide Anion Content Assay Kit (BC1290) were obtained from Solarbio (Beijing, China). CCK-8 (C0041) was purchased from Beyotime Biotechnology (Shanghai, China). SYTO9/PI Live/Dead Bacterial Double Stain Kit was purchased from MaokangBio (MX4234, Shanghai, China). DPBF (D122454) and DCFH-DA (H131224) were purchased from Aladdin (Shanghai, China). The antibodies against F4/80 (GB113373), CD86 (GB150054) and CD206 (GB114055) were obtained from Wuhan Servicebio Technology (Wuhan, China).

### 2.2 Detection of ROS in bacteria

Bacteria were incubated with DHPC (4, 8 μM) and DCFH-DA probe (10 μM) in the dark for 20 min in a total volume of 2 mL, followed by irradiation with a 637 nm laser at a power density of 20 mW/cm^2^ for 10 min (Liu et al., 2025c; Lu et al., 2022). The emission wavelength of the laser (SN240509, Viasho Technology, China) was verified using a fiber-coupled spectrometer (LSA, HighFinesse GmbH, Germany). The power density was calculated by dividing the measured output power (using a laser power meter, S145C/PM100D, Thorlabs, USA) by the spot area, which was determined from the measured spot diameter according to the formula *S* = π*r*^2^. Three successive washes with PBS were then performed on the samples, and ROS generation was measured using flow cytometry (NovoCyte, Agilent, USA). ROS production was further measured using an alternative approach, where 10 μL of stained bacteria was transferred to a glass slide and imaged using fluorescence microscopy (Axio Scope5, ZEISS, Germany).

### 2.3 Bactericidal activity against pathogenic bacteria

To determine the photodynamic bactericidal effect against MRSA or *P. aeruginosa*, bacterial suspension was adjusted to 10^6^ cfu/mL. DHPC was added to the microbial suspension at concentrations ranging from 0 to 20 μM (0, 4, 8, 12, 16, 20 μM). After 20 min of co-incubation, the mixtures were irradiated with light (637 nm, 20 mW/cm^2^, 10 min). Antibacterial efficacy was evaluated by cfu/mL counts on solid medium, with bacterial survival normalized to the DHPC(0 μM) + Dark group as 100% (Zhong et al., 2025).

### 2.4 Live/dead assay of bacteria

MRSA and *P. aeruginosa* were incubated with DHPC at 12 and 16 μM, respectively. Following a 20 min incubation, the suspension was irradiated at 20 mW/cm^2^ for 10 min. Then, 500 μL bacterial suspension in PBS were stained with SYTO9/PI (3.34/20 μM) for 15 min in darkness (Mix et al., 2025). Live (green) and dead (red) bacteria were visualized by placing 10 μL of the stained suspension on a glass slide and imaged via Nikon C2 confocal microscope (Japan).

### 2.5 Detection of cytoplasmic leakage

Bacteria (MRSA or *P. aeruginosa*) grown in LB medium were subjected to three washes with PBS, resuspended in PBS, and incubated with DHPC (12 and 16 μM, respectively; final volume 2 mL) for 20 min. After light irradiation (20 mW/cm^2^, 10 min), centrifugation was carried out (10,000 rpm, 10 min). The supernatant fraction was then harvested. Absorbance at 260 nm of the supernatant was measured to assess cytoplasmic leakage (Zheng et al., 2020).

### 2.6 Biofilm cultivation and bacterial survival assessment

To establish biofilms, bacteria were cultured overnight at 37 °C in culture medium. The overnight culture was diluted 100-fold, and 200 μL aliquots were seeded into glass-bottom 24-well plates. Incubation proceeded at 37 °C for 36 h to promote biofilm development (Rana et al., 2026).

To evaluate the capacity of DHPC-PDT to eradicate microbes within biofilms, the mature biofilms were treated with DHPC at the indicated concentrations and light (20 mW/cm^2^, 10 min). Post-treatment biofilms were washed with PBS, scraped (15 times), and homogenized by pipetting (5 times). Viable colonies were enumerated after serial dilutions were spread onto agar plates and incubated for 24 h at 37 °C. Survival was normalized to DHPC(0 μM) + Dark control (100%) (Manoharan et al., 2020).

### 2.7 Biofilm visualization and quantification

To visually evaluate biofilms, samples were subjected to crystal violet (0.1%) staining for 10 min, rinsed with PBS, and imaged by microscopy. For quantitative assessment, the biofilm samples in 96-well plates were washed three times with 200 μL of PBS, fixed with 200 μL of methanol, air-dried, and stained with 0.1% crystal violet. After washing with PBS, the bound crystal violet was dissolved in 33% acetic acid (200 μL), and the absorbance was measured at 590 nm using a microplate reader (Epoch2, BioTeK, Germany) (Yu et al., 2026).

Biofilm samples in 24-well plates underwent three washes with PBS, and were incubated with 500 μL PBS containing fluorescein diacetate (FDA, 20 μg/mL) for 30 min under darkness. Once rinsed to remove excess dye, confocal images were acquired (Wang et al., 2022).

### 2.8 Animal model and treatment protocols

Male ICR mice (approximately 20.0 g) were purchased from Shanxi Medical University. All animal procedures were performed in accordance with the guidelines of the Laboratory Animal Ethics Committee of Lyuliang University and were approved by the committee (approval no. 2025–0210). After one week of acclimation, a wound (approximately 10 mm) was created on the dorsal side, and 10^7^ MRSA were injected into the wound site to establish infection over 2 days (Pranantyo et al., 2024; Zhang et al., 2023b). Four groups of the infected mice (n = 6 each) were established. Over a 10 day period (on days 0, 2, 4, 6, and 8), mice received subcutaneous treatment every other day as follows: PBS + D, PBS + L (20 mW/cm^2^, 10 min), DHPC (0.5 mg/kg) + D, and DHPC (0.5 mg/kg) + L (20 mW/cm^2^, 10 min). Wound images were taken on alternate days. At day 10, wound samples were harvested, homogenized in PBS, centrifuged (5,000 rpm, 5 min) and diluted. The samples were spread onto agar plates and kept at 37 °C for 24 h to determine the colony count (Chen et al., 2025b).

### 2.9 Histological and immunofluorescence assessment of wound tissues

Paraffin-embedded wound tissues were sectioned at 4 μm after fixation in 4% paraformaldehyde, and then stained with Masson’s trichrome and hematoxylin–eosin (H&E). Additionally, on these sections, immunofluorescence analysis was performed to quantify F4/80, CD86 and CD206 expression levels using corresponding antibodies (GB113373, GB113497, GB114055, Wuhan Servicebio Technology, China) (Chen et al., 2025a; Yang et al., 2023).

### 2.10 Statistical analysis

Statistical evaluation was carried out with SPSS 17.0. All experiments were performed at least three times, and data are expressed as mean ± SD. Statistical significance was determined using two-way ANOVA, Dunnett’s *post-hoc* test or two-way ANOVA, Tukey’s *post-hoc* test, as appropriate. Significance levels are indicated as: ^*^p < 0.05, ^**^p < 0.01, ^***^p < 0.001, and ^****^p < 0.0001; ns, not significant (*p* ≥ 0.05).

## 3 Results

### 3.1 Characterization and ROS generation of DHPC

Given the great potential of DHPC-PDT in antibacterial applications, we aimed to systematically evaluate its efficacy against drug-resistant bacteria. We first synthesized and characterized DHPC, with its synthetic route and chemical structure shown in Figure 1A. The ^1^H NMR, ^13^C NMR and mass spectra of DHPC were presented in Supplementary Figure S1–S3, while its absorption, excitation, and emission spectra were shown in Figures 1B–D. We next examined the ability of DHPC to produce reactive oxygen species (ROS) under light irradiation. As shown in Supplementary Figure S4, light exposure triggered DHPC to produce both Type I ROS, including superoxide anion (O_2_^-•^) and hydroxyl radical (•OH), and Type II ROS, namely singlet oxygen (^1^O_2_). Moreover, upon light irradiation, the UV–vis absorption spectra of DHPC showed a slight decrease in intensity, suggesting favorable photostability (Supplementary Figure S5).

**Figure 1:**
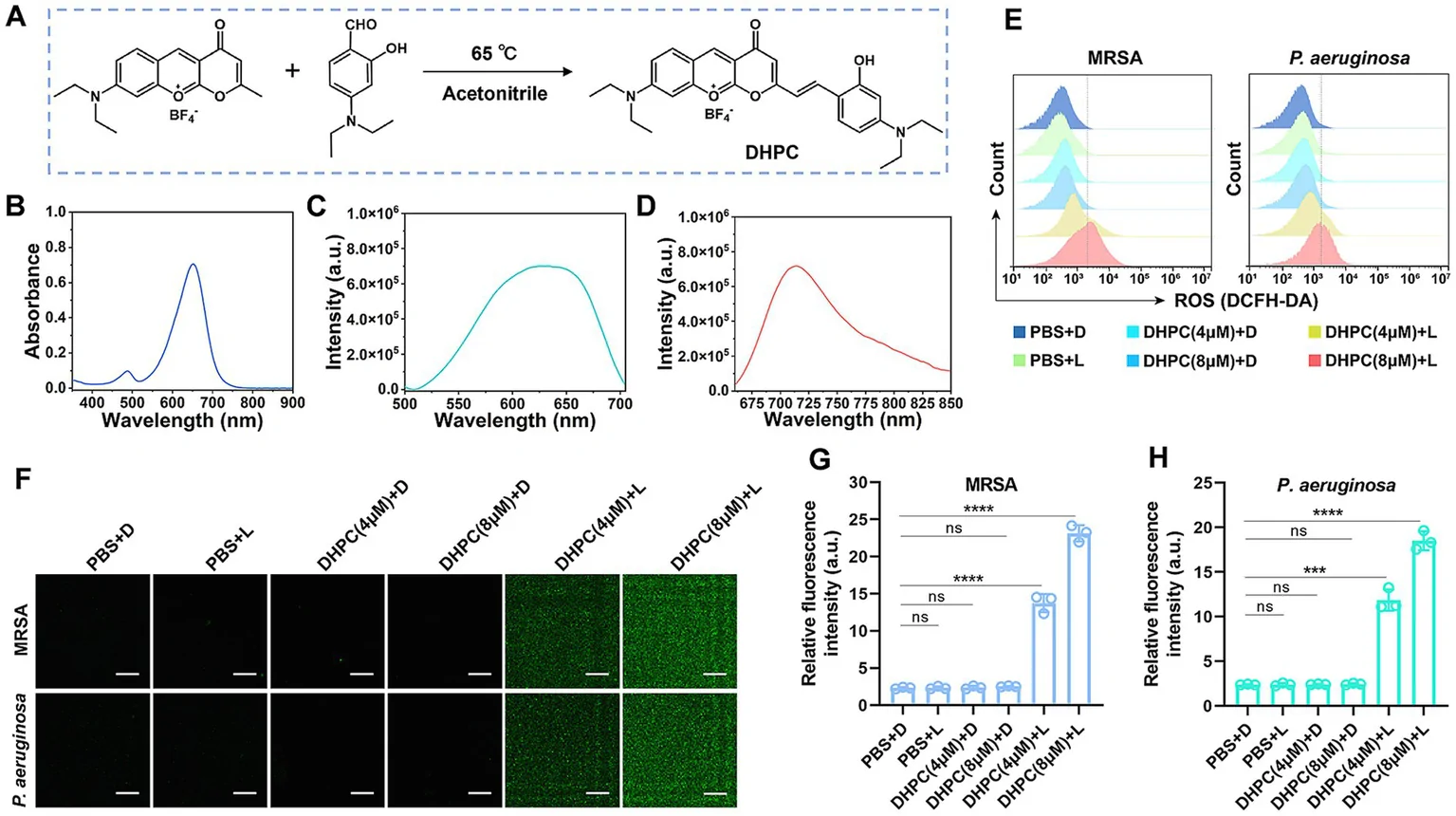
DHPC generates ROS in bacteria upon light irradiation. **(A)** Schematic synthetic route of DHPC. **(B–D)** Absorption, excitation and emission spectra of DHPC (5 μM) in DMSO. **(E)** ROS levels in MRSA and *P. aeruginosa* following DHPC (4, 8 μM) + light (20 mW/cm^2^, 10 min) treatment, measured by DCFH-DA staining and flow cytometric analysis. **(F)** ROS levels in MRSA and *P. aeruginosa* following DHPC (4, 8 μM) + light (20 mW/cm^2^, 10 min) treatment, measured by DCFH-DA staining and fluorescence microscopy. Scale bar = 100 μm. **(G,H)** Fluorescence intensity quantification in (F). Data are presented as mean ± SD (*n* = 3). ^***^p < 0.001, ^****^p < 0.0001, ns, *p* ≥ 0.05 (two-way ANOVA, Dunnett’s *post-hoc* test). D, dark; L, light.

Subsequently, we examined the intracellular ROS generation by DHPC plus light (DHPC+L) within drug-resistant bacteria, including methicillin-resistant *Staphylococcus aureus* (MRSA) and multidrug-resistant *Pseudomonas aeruginosa* (*P. aeruginosa*). Results from flow cytometry combined with the DCFH-DA fluorescent probe showed that DHPC+L induced ROS production within MRSA and *P. aeruginosa*, and a higher DHPC concentration led to greater ROS generation (Figure 1E). Fluorescence imaging and quantitative analysis further confirmed that DHPC+L generated substantial ROS in the two bacterial strains (Figures 1F–H). These results demonstrated the ROS-producing capacity of DHPC+L within bacteria, which is a prerequisite for PDT.

### 3.2 Antibacterial effect of DHPC-PDT against drug-resistant bacteria

The intracellular generation of ROS prompted us to evaluate the antimicrobial efficacy of DHPC-PDT. Bacteria were incubated with varying concentrations of DHPC and then exposed to light. The treated samples were then plated on agar plates and cultured for 24 h. Colony counting assays showed that DHPC-PDT exerted potent bactericidal activity against both MRSA and *P. aeruginosa* in a concentration-dependent manner, with minimum bactericidal concentrations of 16 μM for MRSA and 20 μM for *P. aeruginosa* (Figures 2A–D). To confirm that the observed bactericidal effect was attributable to bacteria-associated DHPC rather than extracellular free DHPC, unbound DHPC was removed by washing prior to light irradiation. As shown in Supplementary Figure S6, notable antibacterial activity persisted after washing, indicating that the photodynamic inactivation primarily originated from bacteria-associated DHPC. Moreover, compared with the commonly used cationic PS methylene blue (MB) (Wang et al., 2023), DHPC-PDT exhibited significantly superior bactericidal efficacy (Supplementary Figure S7). We next employed live/dead staining to further evaluate the bactericidal efficacy of DHPC-PDT. SYTO9 can penetrate intact cell membranes to label live bacteria with green fluorescence, whereas propidium iodide (PI) can only enter membrane-compromised dead cells and stain them with red fluorescence (Mix et al., 2025). As shown in Figure 2E,F, DHPC+L treatment resulted in a marked reduction in green fluorescence and a concomitant increase in red fluorescence, indicating that DHPC-PDT disrupted the bacterial cell membrane, thereby leading to bacterial death. The leakage of bacterial intracellular components following DHPC-PDT treatment further indicated the loss of membrane integrity, providing direct evidence of membrane damage (Figures 2G,H). Given that ATP synthesis in bacteria occurs on the cytoplasmic membrane, we next measured ATP levels following DHPC-PDT treatment (Hurdle et al., 2011). As expected, a pronounced reduction in ATP content was observed (Supplementary Figure S8), further confirming the structural disruption of the bacterial membrane. Collectively, these findings suggested that the bactericidal action of DHPC-PDT was primarily mediated through disruption of the bacterial cell membrane, which was consistent with the membrane-associated distribution of DHPC (Supplementary Figure S9). More importantly, the DHPC dose required for complete bacterial eradication was insufficient to achieve total elimination of tumor cells (Supplementary Figure S10), indicating that DHPC-PDT was a more appropriate therapeutic approach for treating bacterial infections. Furthermore, after daily sublethal DHPC+L exposure for 10 days, the bactericidal efficacy against MRSA and *P. aeruginosa* remained stable, indicating no detectable bacterial resistance (Supplementary Figure S11).

**Figure 2:**
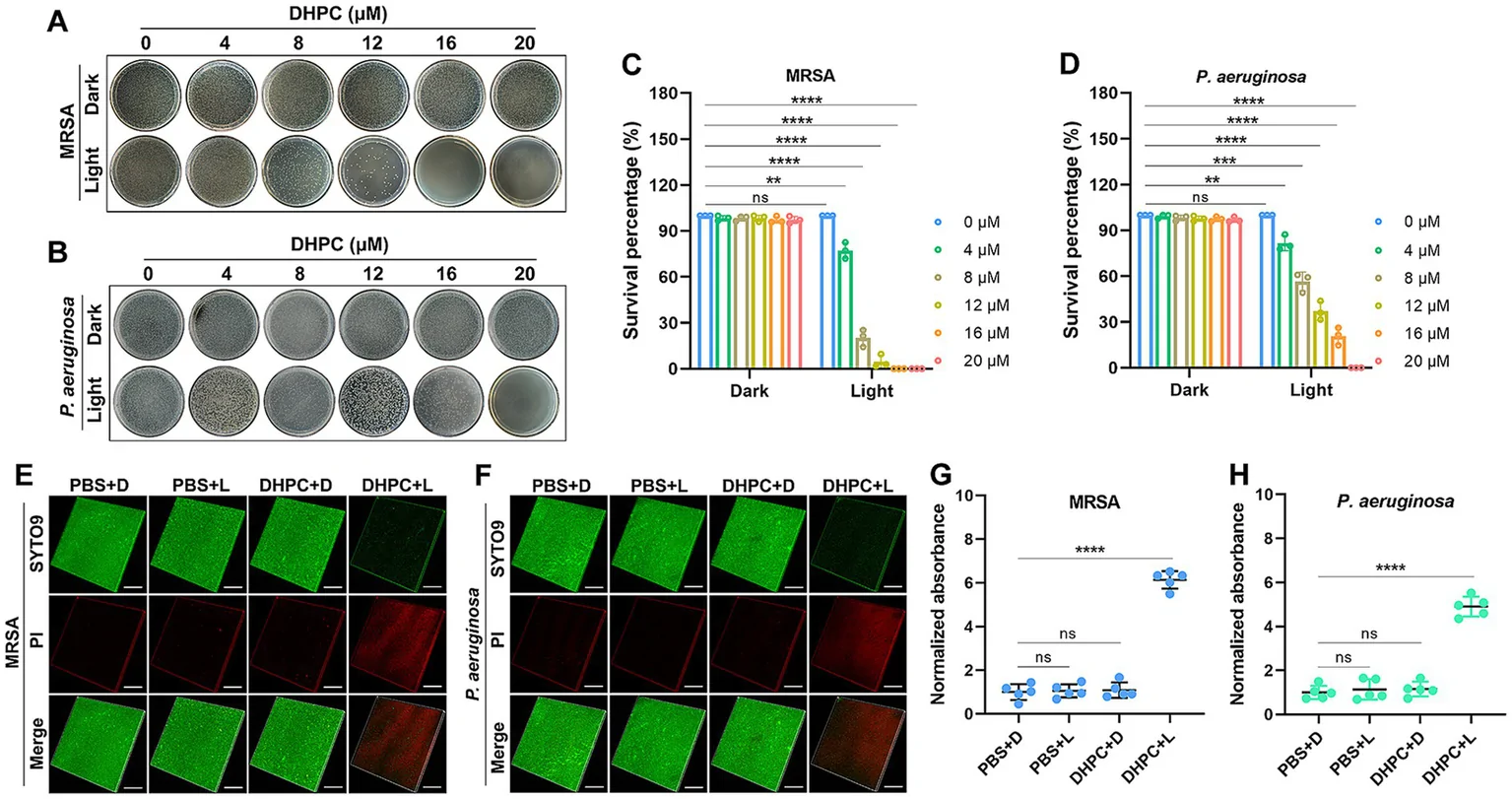
DHPC-PDT kills drug-resistant bacteria. **(A,B)** Representative colony images of MRSA and *P. aeruginosa* after treatment with DHPC (0, 4, 8, 12, 16, 20 μM) + light (20 mW/cm^2^, 10 min). **(C,D)** Survival percentage of bacteria corresponding to the colony counts in (A,B). Data are presented as mean ± *SD* (*n* = 3). ^**^p < 0.01, ^***^p < 0.001, ****p < 0.0001, ns, *p* ≥ 0.05 (two-way ANOVA, Dunnett’s *post-hoc* test). **(E,F)** Confocal microscopy visualization of MRSA and *P. aeruginosa* stained with SYTO9/PI after exposure to DHPC (12 and 16 μM, respectively) + light (20 mW/cm^2^, 10 min). Scale bar = 200 μm. **(G,H)** Absorbance at 260 nm of nucleic acid released from MRSA and *P. aeruginosa* following DHPC (12 and 16 μM, respectively) + light (20 mW/cm^2^, 10 min) treatment. Data are presented as mean ± SD (*n* = 5). ^****^p < 0.0001, ns, *p* ≥ 0.05 (two-way ANOVA, Dunnett’s *post-hoc* test). D, dark; L, light.

### 3.3 Inhibitory effect of DHPC-PDT on biofilm formation

Biofilms play a key role in the pathogenesis of severe infections, making biofilm eradication an important metric for assessing treatment efficacy. Biofilms consist of microorganisms and extracellular polymeric substances (EPS), such as exopolysaccharides, eDNA, and proteins, which collectively impart a net negative charge to the biofilms (Harper et al., 2019; LuTheryn et al., 2023). Thus, cationic PS can accumulate on biofilms through electrostatic interactions and penetrate deeply, enabling effective biofilm elimination upon PDT (Liu et al., 2025b; Wan et al., 2022). Based on these considerations, we investigated the ability of DHPC-PDT to inhibit biofilm formation. Bacteria following DHPC-PDT treatment were cultured for biofilm development, and the biofilm formation was monitored over time. During the initial 12-h formation phase, biofilm attachment was considerably inhibited by DHPC-PDT (Figures 3A,B). At the 36-h maturation phase, quantitative analysis via crystal violet staining showed that in the DHPC+L group, the biofilm biomass was markedly lower than that of the control groups, indicating that DHPC-PDT inhibited biofilm formation (Supplementary Figure S12). Crystal violet staining images also revealed dense biofilm establishment in the control groups, whereas only sparse and punctate biofilms were observed in DHPC+L treated group (Figures 3C,D). Three-dimensional fluorescence images revealed thick biofilm architecture in the control groups, while the DHPC-PDT treated group exhibited scattered and loose structures, suggesting the suppressed biofilm formation following DHPC-PDT treatment (Figure 3E). Taken together, these results demonstrated that DHPC-PDT effectively inhibited biofilm formation.

**Figure 3:**
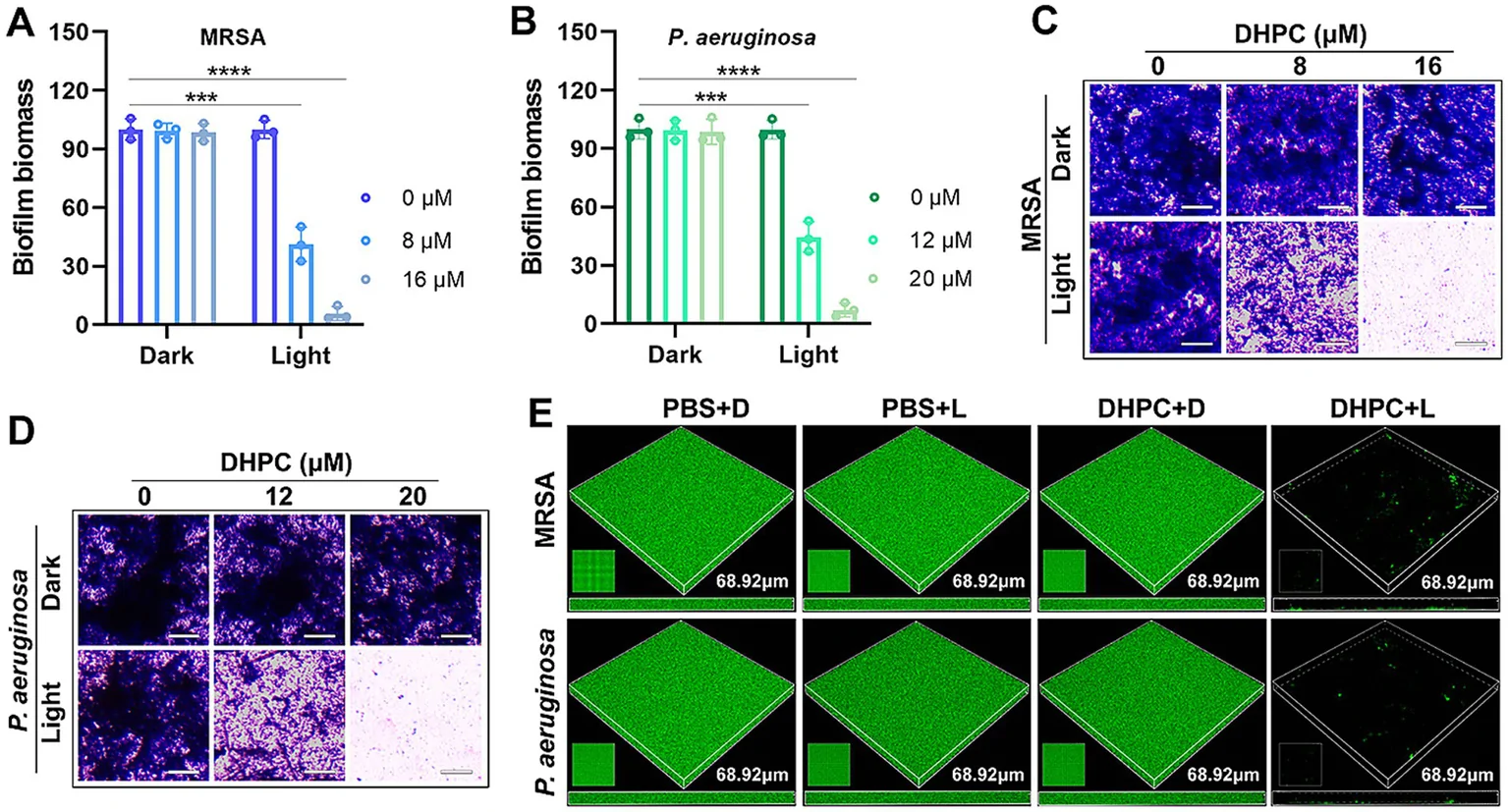
DHPC-PDT inhibits biofilm formation. **(A,B)** Biofilm biomass of MRSA and *P. aeruginosa* after DHPC + light (20 mW/cm^2^, 10 min) treatment followed by 12 h of incubation. Data are presented as mean ± SD (*n* = 3). ^***^p < 0.001, ^****^p < 0.0001 (two-way ANOVA, Dunnett’s *p*ost-*hoc* test). **(C,D)** Biofilm images of MRSA and *P. aeruginosa* after DHPC + light (20 mW/cm^2^, 10 min) treatment followed by 36 h of incubation for biofilm formation and crystal violet staining. Scale bar = 100 μm. **(E)** Confocal visualization of MRSA and *P. aeruginosa* biofilms formed after DHPC (16 and 20 μM, respectively) + light (20 mW/cm^2^, 10 min) treatment and 36 h of subsequent incubation. D, dark; L, light.

### 3.4 Disruption of mature biofilms by DHPC-PDT

The inhibition of biofilm formation and the disruption of mature biofilms involve different mechanisms. For mature biofilm disintegration, PS needs to penetrate the biofilms to kill sheltered bacteria and remove the extracellular matrix. As shown in Supplementary Figure S13, DHPC was able to effectively penetrate and accumulate in the deep layers of the biofilms. To assess the capacity of DHPC-PDT against mature biofilms, we exposed pre-formed biofilms to DHPC-PDT and examined its effects on embedded bacteria and biofilm integrity. As shown in Figures 4A–D, DHPC-PDT exhibited a concentration-dependent bactericidal effect against bacteria embedded within biofilms, with superior efficacy to that of MB-PDT (Supplementary Figure S14). The disruptive effect of DHPC-PDT on mature biofilms was evaluated from three complementary perspectives. Quantitatively, crystal violet staining revealed that DHPC-PDT treatment markedly reduced total biofilm biomass compared with the control groups (Figures 4E,F), with a greater reduction than that achieved by MB-PDT (Supplementary Figure S15). Visually, crystal violet staining images showed solid and confluent biofilm coverage in the control groups, whereas only residual and punctate biofilms were observed following DHPC-PDT treatment (Figure 4G). Structurally, fluorescein diacetate-stained three-dimensional images revealed thick and compact biofilm architecture in the control groups. In contrast, the DHPC-PDT treated group exhibited thin and scattered structures (Figure 4H). Collectively, these results demonstrated that DHPC-PDT could disrupt mature biofilms, underscoring its promise for biofilm eradication.

**Figure 4:**
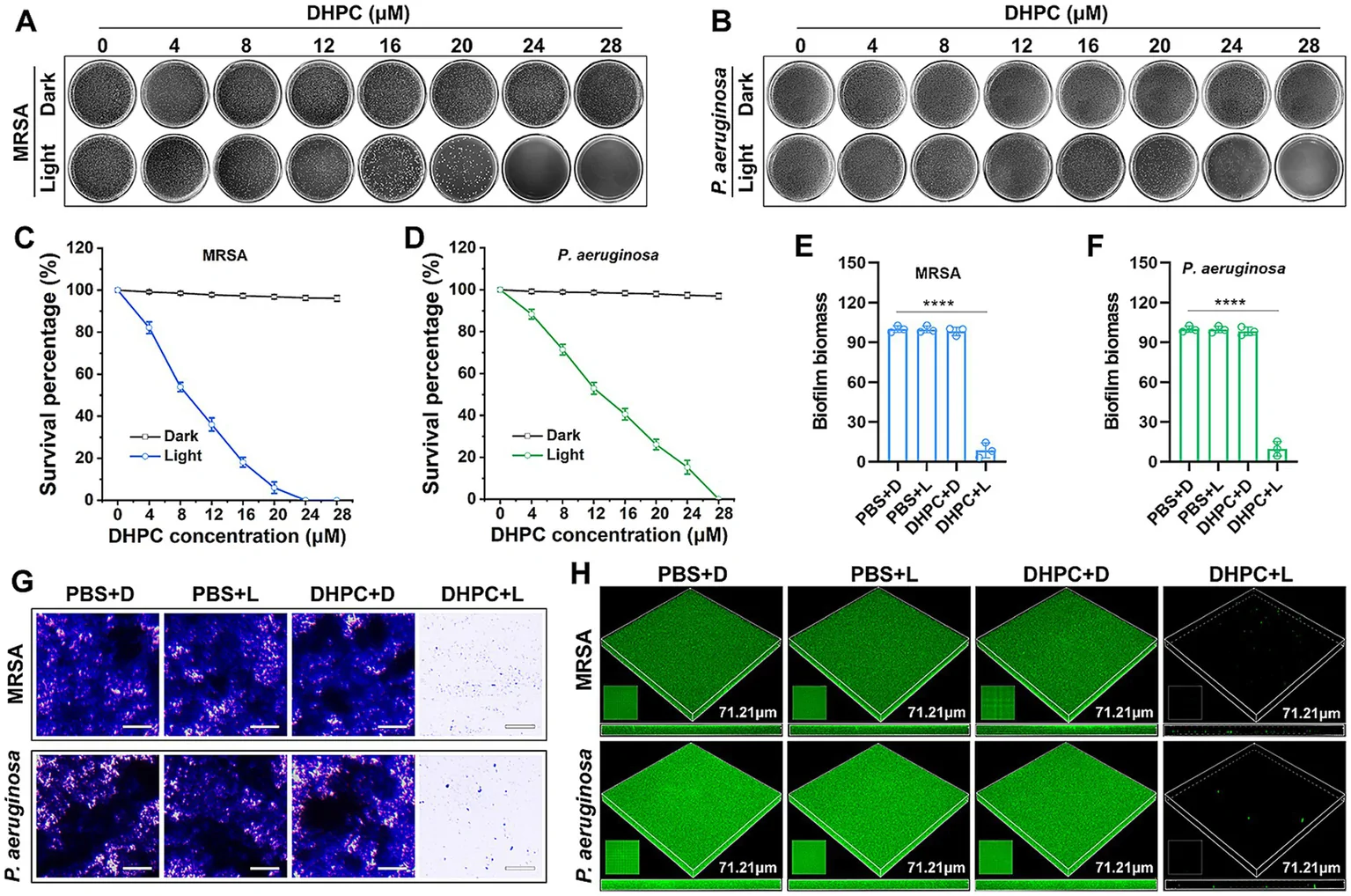
DHPC-PDT eradicates biofilm-embedded bacteria and disrupts biofilms. **(A,B)** Representative colony images of MRSA and *P. aeruginosa* isolated from 36 h-old biofilms after DHPC (0, 4, 8, 12, 16, 20, 24, 28 μM) + light (20 mW/cm^2^, 10 min) treatment. **(C,D)** Survival percentage of bacteria corresponding to the colony counts in (A, B). Data are presented as mean ± *SD* (*n* = 3). **(E,F)** Biofilm biomass of mature MRSA and *P. aeruginosa* biofilms after DHPC (24 and 28 μM, respectively) + light (20 mW/cm^2^, 10 min) treatment. Data are presented as mean ± *SD* (*n* = 3). ^****^p < 0.0001 (two-way ANOVA, Dunnett’s *post-hoc* test). **(G)** Images of MRSA and *P. aeruginosa* biofilms stained with crystal violet after treatment with DHPC (24 and 28 μM, respectively) + light (20 mW/cm^2^, 10 min). Scale bar = 100 μm. **(H)** Confocal visualization of MRSA and *P. aeruginosa* biofilms after treatment with DHPC (24 and 28 μM, respectively) + light (20 mW/cm^2^, 10 min). D, dark; L, light.

### 3.5 Promotion of wound healing and alleviation of inflammation by DHPC-PDT

To further validate its therapeutic potential, we evaluated the ability of DHPC-PDT to eradicate drug-resistant bacteria *in vivo* using the method illustrated in Figure 5A (Cao et al., 2019; Zhang et al., 2023a). The wound retention and biodistribution of DHPC were first assessed. As shown in Figure 5B, DHPC persisted at the wound site for up to 4 h, decreased markedly at 8 h, and was largely cleared by 10 h. Accordingly, subsequent DHPC-PDT was performed at 1 h post-injection. *Ex vivo* imaging and quantitative analysis revealed negligible DHPC signal in major organs and blood (Figures 5C,D), indicating that only minimal DHPC entered the circulatory system via the wound site, which was consistent with previous studies (Li et al., 2017; Peng et al., 2022; Vairo et al., 2019).

**Figure 5:**
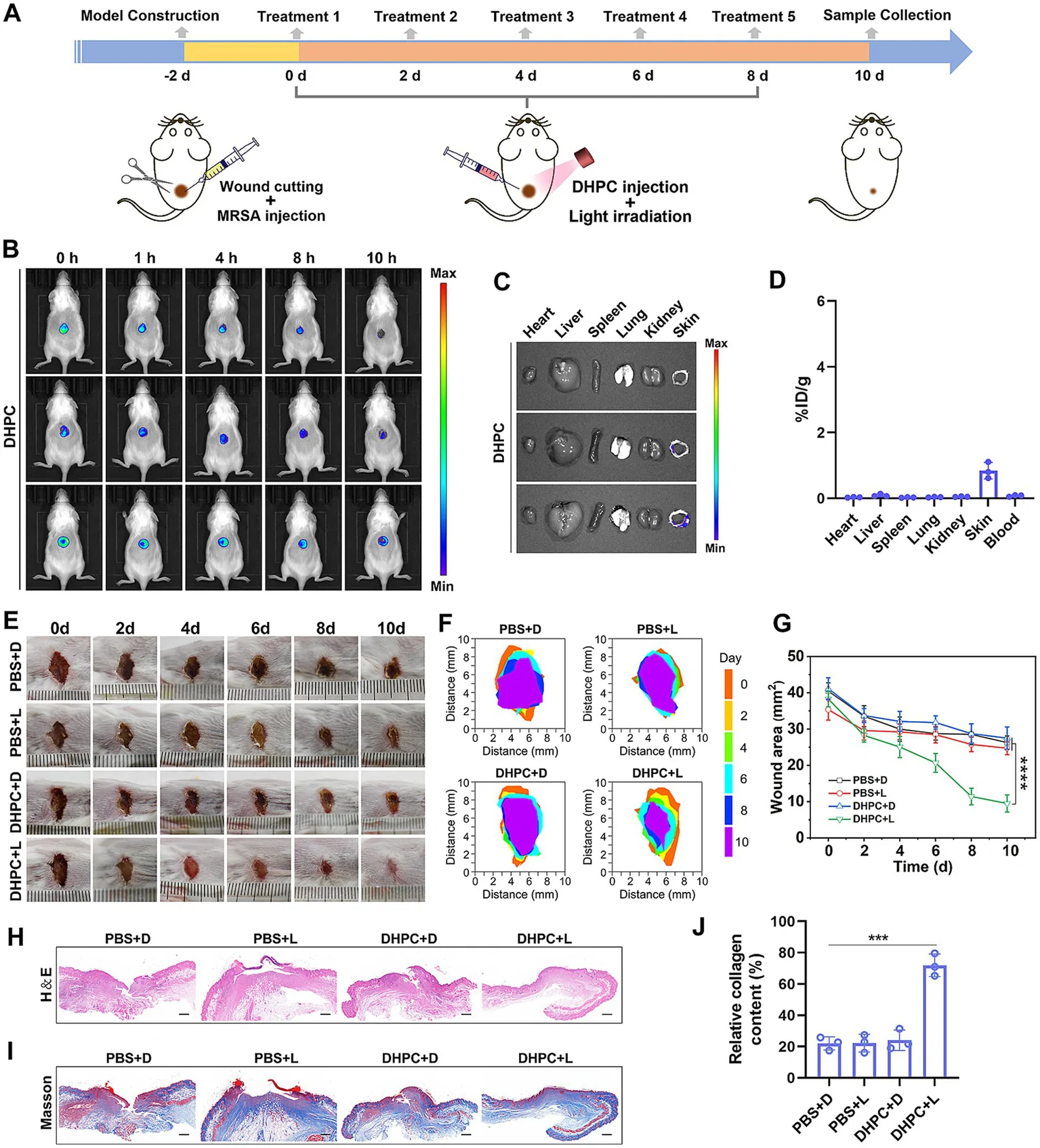
DHPC-PDT promotes wound healing. **(A)** Description of the mouse wound infection model and the DHPC-PDT protocol. **(B)** *In vivo* fluorescence imaging of mice at 0, 1, 4, 8, and 10 h post DHPC injection. **(C)** *Ex vivo* visualization of DHPC in key organs 10 h after injection. **(D)** DHPC levels in major organs as well as blood at 10 h following injection. Data are presented as mean ± *SD* (*n* = 3). **(E,F)** Appearance of wounds on mice subjected to PBS+D, PBS+L, DHPC+D, and DHPC+L treatments. **(G)** Wound area in mice following treatment. Data are presented as mean ± *SD* (*n* = 6). ^****^p < 0.0001 (two-way ANOVA, Dunnett’s *post-hoc* test). **(H,I)** Representative histological images of H&E and Masson’s trichrome staining of skin wound tissue sections. Scale bar = 500 μm. **(J)** Quantification of collagen deposition based on Masson’s trichrome staining. Data are presented as mean ± *SD* (*n* = 3). ^***^p < 0.001 (two-way ANOVA, Dunnett’s *post-hoc* test). D, dark; L, light.

Subsequently, a wound infection model was established by injecting MRSA into the wound site, after which DHPC-PDT treatment was administered. Compared with the control groups, DHPC-PDT accelerated wound healing, with marked wound closure observed by day 6 and nearly complete healing achieved by day 10 post-treatment (Figures 5E–G). Histological analysis via H&E staining revealed that the control groups exhibited incomplete re-epithelialization with discontinuous or absent epidermal coverage over the wound surface, whereas the DHPC+L group showed complete and continuous re-epithelialization with well-structured epidermal layers, further confirming the superior wound healing effect of DHPC-PDT (Figure 5H). Furthermore, Masson’s trichrome staining and quantitative analysis revealed increased blue-stained collagen deposition at the wound site in the DHPC+L group compared to the controls, providing additional evidence for the healing efficacy of DHPC-PDT (Figures 5I,J) (Zhang et al., 2026). Collectively, these findings indicated that DHPC-PDT effectively improved wound healing.

Wound healing is closely associated with bacterial clearance and host immune regulation. Therefore, we examined the bacterial burden and inflammatory levels in wounds following DHPC-PDT treatment. As shown in Figure 6A,B, DHPC-PDT treatment markedly reduced the bacterial load at the wound site, with a concurrent decrease in the levels of inflammatory cytokines including tumor necrosis factor-α (TNF-α) and interleukin-6 (IL-6) (Figures 6C,D). Furthermore, the phenotypic switching of macrophages toward an anti-inflammatory phenotype, as evidenced by increased infiltration of M2 macrophages (F4/80^+^ and CD206^+^) and decreased infiltration of M1 macrophages (F4/80^+^ and CD86^+^) in the wound tissue (Figure 6E), further supports the immunomodulatory role of DHPC-PDT in the wound healing process (Li et al., 2026). Taken together, these results demonstrated that DHPC-PDT effectively eliminated bacteria at the wound site and attenuated local inflammation, thereby promoting wound healing.

**Figure 6:**
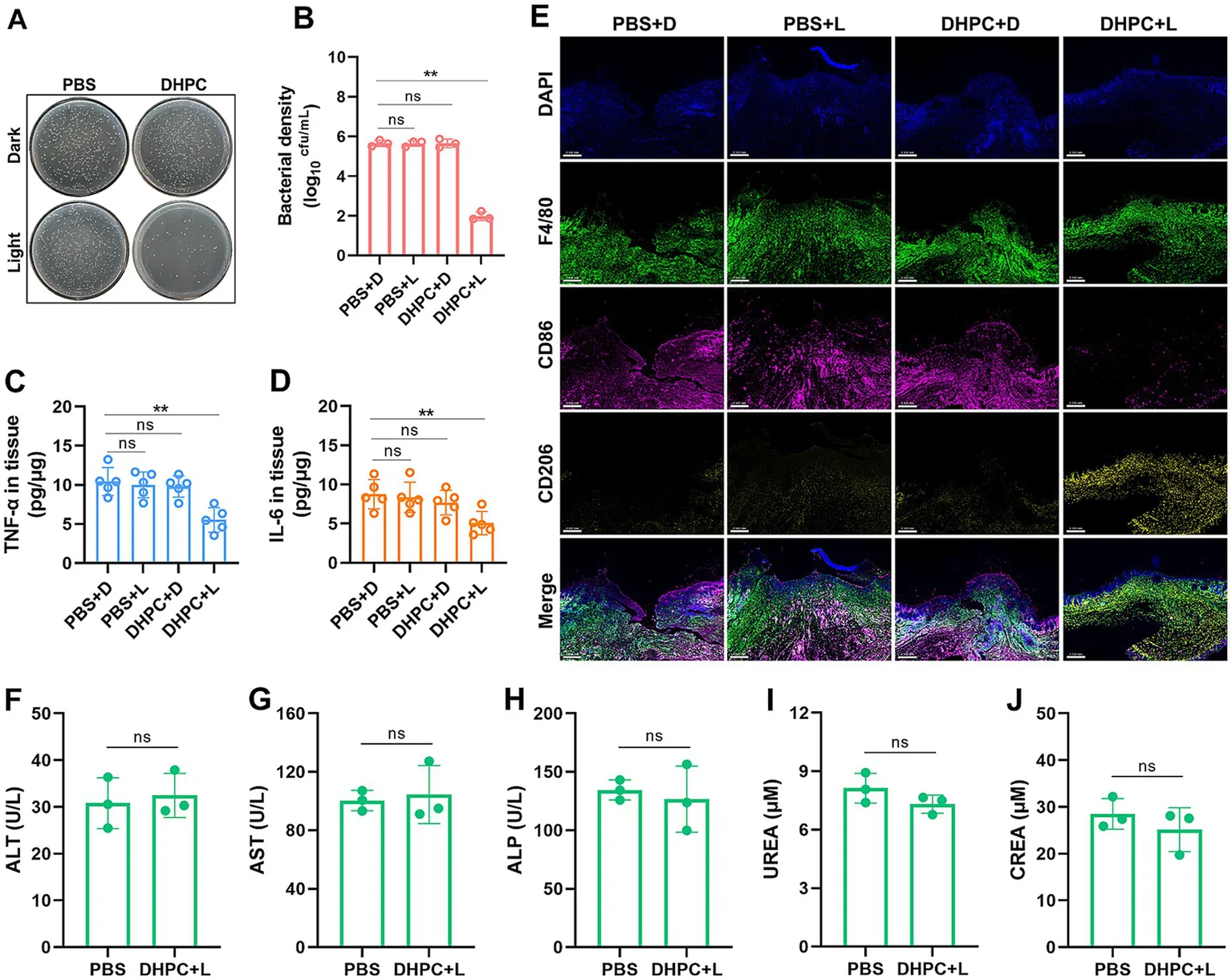
DHPC-PDT eliminates bacteria and attenuates inflammation at the wound site. **(A)** Representative images of bacterial colonies derived from wound samples. **(B)** Viable bacterial quantification in wound tissues. Data are presented as mean ± SD (*n* = 3). ^**^p < 0.01, ns, *p ≥* 0.05 (two-way ANOVA, Dunnett’s *post-hoc* test). **(C,D)** TNF-α and IL-6 levels detected in treated skin tissues. Data are presented as mean ± SD (n = 5). ^**^p < 0.01, ns, *p* ≥ 0.05 (two-way ANOVA, Dunnett’s *p*ost-*hoc* test). **(E)** Representative immunofluorescence micrographs of treated wound tissues labeled with F4/80 (green), CD86 (purple), CD206 (yellow), and DAPI (blue). Scale bar = 500 μm. **(F-J)** Serum biochemical marker levels in mice at 10 d. Data are presented as mean ± SD (*n* = 3). ns, *p* ≥ 0.05 (two-way ANOVA, Tukey’s *post-hoc* test). D, dark; L, light.

After treatment, we assessed the biocompatibility of DHPC in mice. Throughout the entire treatment period, the body weight of mice remained stable without apparent fluctuations (Supplementary Figure S16A). Histological assessment of major organs revealed no microscopic abnormalities (Supplementary Figure S16B). DHPC also exhibited low hemolytic activity, with a hemolysis rate below 5% at the concentration of 50 μM (Supplementary Figure S16C) (Ullah et al., 2024). At day 10 (2 days after the final treatment), no obvious changes were observed in key serum biochemical markers (Figures 6F–J) or major hematological parameters (Supplementary Figure S17). To further evaluate its biosafety, we extended the observation period to 14 days post-injection (Liu et al., 2020). Consistent with the 10-day results, no body weight loss was observed during the treatment period with monitoring every 2 days (Supplementary Figure S18A). Key serum biochemical and hematological parameters remained within normal ranges at day 14 (Supplementary Figure S18B-L). Histological examination of major organs via H&E staining showed no discernible pathological lesions (Supplementary Figure S18M). Taken together, these findings preliminarily demonstrated that DHPC possesses good biocompatibility.

## 4 Discussion

### 4.1 Bactericidal activity against gram-positive and gram-negative bacteria

The surfaces of both gram-positive and gram-negative bacteria are negatively charged (Lin et al., 2018). This property allows cationic PS to be enriched through electrostatic interactions, thereby promoting their uptake into the cells and enhancing the photodynamic bactericidal efficacy. Our results revealed that cationic PS DHPC-mediated PDT exhibited remarkable bactericidal effects against both MRSA (gram-positive bacteria) and *P. aeruginosa* (gram-negative bacteria). However, higher concentrations of DHPC were needed for the eradication of *P. aeruginosa* (Figures 2A–D). This was mainly because *P. aeruginosa* possessed a dense outer membrane that increased the difficulty of entry for PS, thus requiring higher concentrations to overcome the barrier (Heesterbeek et al., 2019). It is worth noting that the surface charge properties of fungal cells are more complex, and their interaction with cationic PS may differ from that of bacteria (Gibała et al., 2021; Nosanchuk and Casadevall, 1997). Given the effective elimination of both types of bacteria, subsequent studies may further explore the bactericidal effects and mechanisms of DHPC-PDT against fungi, such as *Candida albicans* and *Candida glabrata*, to comprehensively evaluate its broad-spectrum antimicrobial potential.

### 4.2 Eradication of embedded bacteria and disruption of biofilms

Biofilms provide a protective barrier for embedded bacteria, shielding them from antibiotics and other antimicrobial agents, which poses a marked obstacle to conventional treatment. PDT, especially cationic PS-mediated PDT, has emerged as a promising strategy for biofilm eradication. Biofilms are composed of a complex array of components, including extracellular polysaccharides, proteins, lipids, nucleic acids, and other macromolecules. These constituents collectively impart a negative charge to the matrix, which enables cationic PS to be recruited via electrostatic attraction and achieves localized enrichment (Harper et al., 2019; LuTheryn et al., 2023). This enrichment enhances the photodynamic efficacy in both disrupting the biofilm matrix and eliminating the resident bacteria. Our studies demonstrated that the cationic PS DHPC-mediated PDT possessed remarkable biofilm disintegration efficacy. Specifically, DHPC-PDT not only disrupted the structural integrity of the biofilms by degrading extracellular matrix components, but also eliminated bacterial populations residing in the deeper layers of the biofilms (Figure 4). Moreover, DHPC-PDT markedly inhibited biofilm formation (Figure 3), thereby preventing biofilm regeneration. Collectively, these findings indicated that DHPC-PDT overcame the limitations of conventional antibiotics, which typically exhibited poor efficacy against persister bacteria. Therefore, DHPC-PDT may represent a potential therapeutic approach for biofilm-associated infectious diseases.

### 4.3 Biodistribution and biosafety assessment

Previous studies have reported that administration through wounded skin generally results in very limited systemic drug absorption (Li et al., 2017; Peng et al., 2022; Vairo et al., 2019). Our findings are consistent with these observations. *Ex vivo* imaging and quantitative analyses revealed that no appreciable DHPC signal was detected in the major organs, including the heart, liver, spleen, lung, and kidney, nor in the blood following wound administration (Figures 5C,D). These findings suggested negligible systemic absorption of DHPC via the wound site. Furthermore, *in vivo* imaging showed that the amount of DHPC at the wound site gradually decreased over time, with the majority being cleared by 10 h post-administration (Figure 5B). This suggested a low propensity for long-term accumulation of DHPC in local tissues and thus a reduced risk of local toxicity.

In terms of biosafety evaluation, we systematically assessed a range of parameters after DHPC-PDT treatment. No statistically significant abnormalities were observed in serum biochemical (including liver and renal function markers) or hematological parameters (Figures 6F–J and Supplementary Figure S17) (Liu et al., 2020). Mouse body weights maintained a normal increasing trend throughout the treatment period, with no notable differences compared to the control groups (Supplementary Figure S16A). Histopathological examination of major organs, including the heart, liver, spleen, lung, and kidney, following DHPC-PDT treatment revealed no obvious tissue damage (Supplementary Figure S16B). Concurrently, *in vitro* hemolysis assay revealed that the hemolysis rate of DHPC remained below 5% within the tested concentration range (Supplementary Figure S16C). Collectively, these results indicated that DHPC exhibits favorable biosafety under topical wound administration conditions.

### 4.4 Advantages and novelty of DHPC-PDT as an antibacterial strategy

Cationization represents an effective strategy to promote the uptake of PS by bacteria. Various cationic PS systems have been developed, including polymer-based, peptide-based, nanoparticle-based, and small-molecule platforms (Liu et al., 2025a; Lyu et al., 2024). However, owing to the high density of positive charges, cationic polymers and nanoparticles are often associated with severe toxicity, as well as complex synthesis and batch-to-batch variability. In the case of nanoparticles, their relatively large dimensions further restrict penetration into bacteria and dense biofilm matrices. Cationic peptides, on the other hand, are frequently compromised by enzymatic degradation and poor stability in physiological environments (Bicker and Cobb, 2020; Ghosh et al., 2024). Thus, despite the extensive development of various PS systems, small molecules have consistently remained the focus of PS development (Wang et al., 2026). Cationic small-molecule PS possess distinct advantages, including effective biofilm penetration and bacterial entry due to their small size, excellent stability against degradation, straightforward and reproducible synthesis, and, more importantly, moderate positive charges that are not associated with severe toxicity (Amorim et al., 2025). Nevertheless, the cationic small-molecule PS reported to date still face certain limitations. For instance, porphyrin-based PS are typically activated by short-wavelength light, which limits tissue and biofilm penetration (Silva et al., 2024). Some small-molecule PS rely on the oxygen-dependent Type I mechanism, which severely restricts their efficacy in hypoxic environments (Almodôvar et al., 2025; Maisch et al., 2014). In other cases, the antibacterial activity of the PS is inherently inadequate. Therefore, there is an urgent need to develop novel cationic small-molecule PS for combating drug-resistant bacteria. Our results demonstrated that DHPC could enter drug-resistant bacteria and generate ROS via both Type I and Type II photodynamic pathways to kill bacteria (Figures 1E–H and Supplementary Figure S4). Moreover, DHPC was capable of penetrating biofilms and, upon red-light excitation, eradicating both the embedded bacteria and the biofilm matrix (Figure 4). More importantly, DHPC-PDT showed superior efficacy over MB (currently in clinical development) in eradicating planktonic bacteria, biofilm-embedded bacteria, and biofilm matrix (Supplementary Figures S7, S14 and S15). Furthermore, preliminary experiments indicated that DHPC possessed favorable biocompatibility. Therefore, compared with existing cationic PS systems, DHPC-PDT exhibits a distinct set of features that offer advantages for antibacterial applications.

We introduced a novel molecular scaffold into the antibacterial PDT field. This scaffold offers a potential solution to the scarcity of PS and opens avenues for developing novel antibacterial PS. DHPC possesses multiple modifiable sites, which allow for future rational modifications to enhance its bactericidal efficacy and biosafety. Moreover, this structural flexibility also enables its integration with nanoplatforms for the development of novel cationic nanosystems.

It is noteworthy that our study is not simply an extension of DHPC application from antitumor to antibacterial fields. Rather, guided by structure-based considerations, we identified a more suitable application for DHPC-PDT, aligning with the concept of drug repurposing. Indeed, DHPC-PDT exhibited superior antibacterial activity against drug-resistant bacteria over its antitumor efficacy (Figures 2A–D and Supplementary Figure S10). Moreover, the antibacterial mechanism of DHPC-PDT, which involves bacterial membrane disruption, ATP synthesis impairment, and leakage of cellular contents, is distinct from its mitochondria-targeting antitumor mechanism. Therefore, our study possesses both mechanistic and pharmacological novelty.

## 5 Conclusion

In conclusion, prompted by the intrinsic positive charges of DHPC and its promising properties, we investigate the effects of DHPC-PDT against drug-resistant bacteria and biofilms (Figure 7). Our results show that DHPC-PDT dramatically eliminates drug-resistant pathogens, including gram-positive (MRSA) and gram-negative (multidrug-resistant *P. aeruginosa*) bacteria, with superior efficacy compared to MB-PDT. Moreover, serial subculturing in the presence of sublethal concentrations of DHPC does not induce detectable resistance. Further studies demonstrate that DHPC-PDT effectively inhibits biofilm formation, eradicates mature biofilms, and eliminates drug-resistant bacteria within the biofilms. *In vivo* experiments reveal that DHPC-PDT clears wound bacteria, reduces local inflammation, and facilitates the repair of infected wounds. In addition, DHPC exhibits acceptable biosafety *in vivo*. Overall, DHPC emerges as a potential PS for countering drug-resistant bacterial infections.

**Figure 7:**
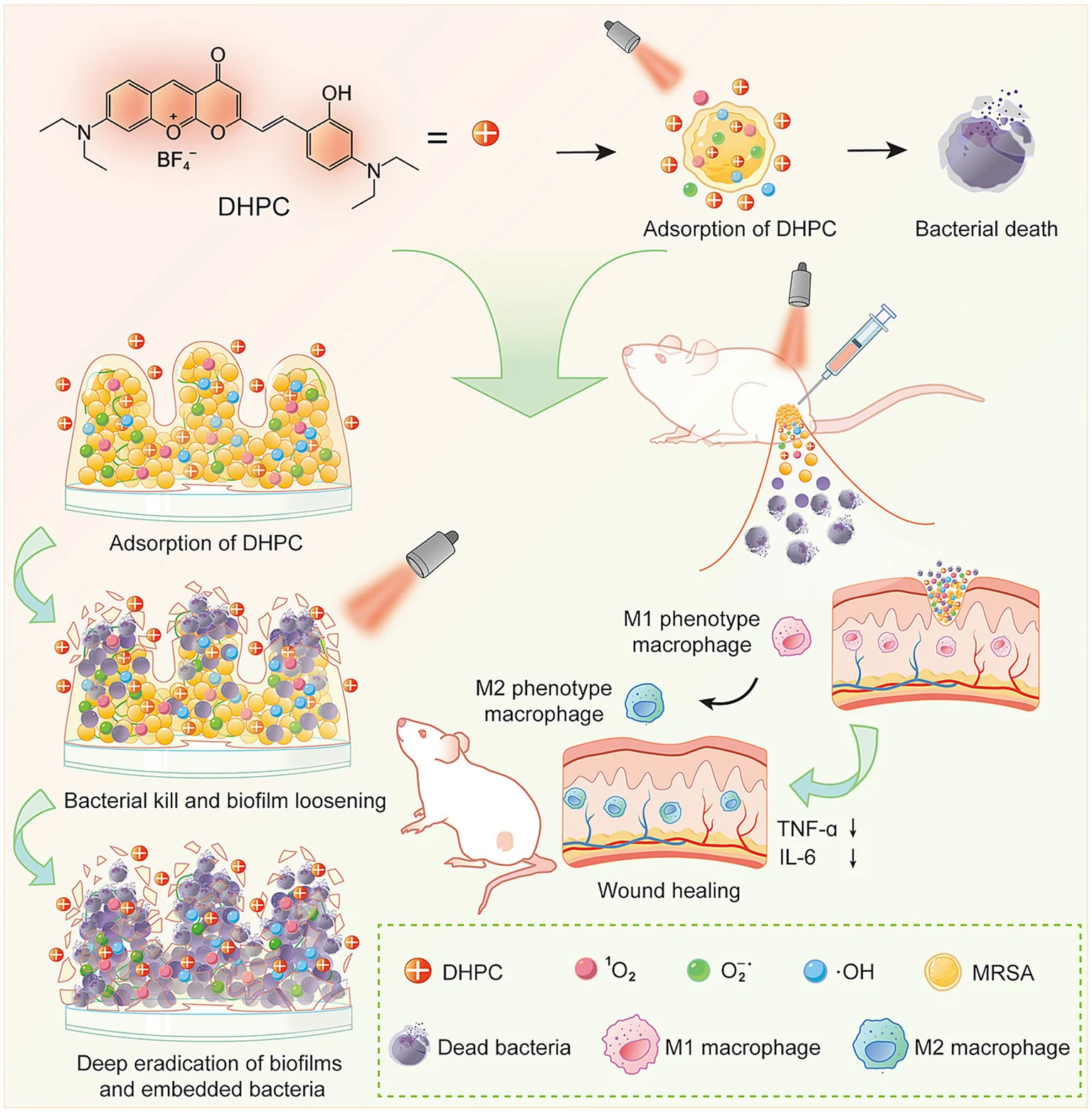
The cationic PS DHPC accumulates on the surfaces of negatively charged bacteria and biofilms. Upon light irradiation, DHPC generates ROS to eradicate drug-resistant bacteria and biofilms. Moreover, DHPC-PDT clears wound bacteria, reduces inflammation, and accelerates the healing of infected wounds, suggesting its potential as an effective therapeutic strategy against drug-resistant bacterial infections.

## Data Availability

The original contributions presented in the study are included in the article/Supplementary material, further inquiries can be directed to the corresponding authors.
