# Supplementary material for: Advanced applications of cationic photosensitizer DHPC in photodynamic eradication of drug-resistant bacteria
Source: Front Microbiol. 2026 Sep 22;17:1925192. doi: 10.3389/fmicb.2026.1925192 (PMC13638522; doi:10.3389/fmicb.2026.1925192)
Supplement: Supplementary file 1 [file Data_Sheet_2.docx]

Supplementary Material

# 1 Supplementary Materials and Methods

## 1.1 Synthesis of compound DHPC

0.185 mg of **a** (coumarin compound) and 0.2775 mg of **b** (4-diethylaminosalicylaldehyde) (1:1.5 molar ratio) were dissolved in acetonitrile. 40 μL of piperidine were added, and the mixture was heated under reflux at 65 °C for 8 hours. After the reaction, a solid precipitated out. The solid was collected by suction filtration, and the filter cake was recrystallized from acetonitrile to obtain the target compound **DHPC**. ^1^H NMR (400 MHz, DMSO) *δ* 10.51 (s, 1H), 8.70 (s, 1H), 8.20 (d, *J* = 15.0 Hz, 1H), 7.78 (d, *J* = 9.1 Hz, 1H), 7.64 (d, *J* = 9.0 Hz, 1H), 7.27 (s, 1H), 6.86 (d, *J* = 9.1 Hz, 1H), 6.80 (d, *J* = 15.1 Hz, 1H), 6.63 (s, 1H), 6.37 (d, *J* = 9.2 Hz, 1H), 6.19 (s, 1H), 3.54 (q, *J* = 7.1 Hz, 4H), 3.42 (q, *J* = 7.1 Hz, 4H), 1.16 (q, *J* = 7.2 Hz, 12H). ^13^C NMR (101 MHz, DMSO) *δ* 178.49, 161.36, 158.62, 157.83, 153.68, 153.16, 146.37, 132.71, 110.76, 108.65, 108.42, 105.94, 99.24, 96.58, 95.92, 44.63, 44.28, 38.90, 12.61, 12.37. HR-MS: calculated for C_28_H_31_N_2_O_4_^+^ 459.2278 [M]^+^, found 459.2269 [M]^+^.

## 1.2 Singlet oxygen (^1^O_2_), superoxide anion (O_2_^−•^) and hydroxyl radical (•OH) detection

Singlet oxygen (^1^O_2_) assay. DHPC (8 μM) and 1,3-Diphenylisobenzofuran (DPBF, OD = 1) were mixed and irradiated (637 nm; 20 mW/cm^2^, 0, 2.5, 5, 7.5, 10 min). Singlet oxygen generation was assessed by measuring DPBF absorbance at 411 nm using a UV-Vis spectrophotometer (UV-3600i Plus, Shimadzu, Japan) (Qi et al., 2020).

Superoxide anion (O_2_^−•^) assay. DHPC (8 μM) solutions were irradiated (637 nm, 20 mW/cm^2^) for 0, 2.5, 5, 7.5, or 10 min. Superoxide anion generation was assessed using a Superoxide Anion Content Assay Kit (BC1290, Solarbio). The product absorbance was recorded at 530 nm using UV-Vis spectrophotometry.

Hydroxyl radical (•OH) assay. DHPC (8 μM) and hydroxyphenyl fluorescein (HPF, 10 μM) were mixed and irradiated (637 nm, 20 mW/cm^2^, 0, 2.5, 5, 7.5, 10 min). Hydroxyl radical generation was assessed using a fluorescence spectrophotometer (FLS1000, Edinburgh instruments, UK) (Zhu et al., 2025).

## 1.3 ATP level assay

MRSA and *P. aeruginosa* were treated with 12 μM and 16 μM DHPC, respectively, for 20 min, followed by irradiation with 637 nm light at 20 mW/cm^2^ for 10 min. After these treatments, ATP levels were measured using an ATP assay kit (BC0305, Solarbio). ATP values were normalized to the PBS+D control (100%).

## 1.4 4T1 cell viability assay

4T1 cells (mouse mammary carcinoma cells) were plated in 96-well plates at a density of 5000 cells per well and incubated overnight (12 h) at 37°C in a 5% CO_2_ atmosphere. Subsequently, the culture medium was exchanged with 100 μL of fresh DHPC supplemented medium, with final concentrations of 20 μM. After 20 min of incubation, cells were exposed to light (20 mW/cm^2^, 10 min) and further maintained for 24 h. Afterward, each well received 10 μL of CCK-8, and the plates were incubated for 2 h, with the absorbance then being read at 450 nm. Cell viability was normalized to the PBS+D group (set as 100%).

## 1.5 In vivo biodistribution study

To evaluate the tissue distribution of DHPC, a subcutaneous injection of DHPC (0.5 mg/kg) was administered into the infected area of mice. The diffusion of DHPC was monitored using an animal live imaging system (IVIS Lumina LT Series Ⅲ, Revvity, Shanghai, China) at 0, 1, 4, 8, and 10 h post-injection. Blood samples and major organs (heart, liver, spleen, lung, kidney, and skin) were collected. These tissues were subsequently subjected to dual analyses, ex vivo fluorescence imaging to visualize DHPC localization, and quantitative fluorometric assessment. For the latter, each organ was weighed and processed by homogenization in PBS (1:9, w/v). Following centrifugation (3170×g, 20 min), the fluorescence intensity of the supernatant was quantified via a spectrophotometer (Synergy H1, BioTek Instruments, USA). The biodistribution data were then quantified and expressed as %ID/g (the percentage of injected dose per gram of tissue) (Li et al., 2022; Li et al., 2017; Su et al., 2024).

## 1.6 Blood biochemistry and hematology

Blood was obtained from the retro-orbital plexus on day 10, two days following the final treatment, using potassium EDTA tubes for hematological analysis and drug-free tubes for serum biochemistry. Whole blood (100 µL) in EDTA tubes was used for hematological analysis. For serum biochemistry, 900 µL of blood was centrifuged (3500 rpm, 12 min) to obtain at least 300 µL of plasma. Assessed parameters included liver and kidney function markers (ALT, alanine aminotransferase; AST, aspartate aminotransferase; ALP, alkaline phosphatase; UREA; CREA, creatinine), and hematological parameters (WBC, white blood cell; LY, lymphocyte; RBC, red blood cell; HGB, hemoglobin; HCT, hematocrit; MCV, mean corpuscular volume; MCH, mean corpuscular hemoglobin; MCHC, mean corpuscular hemoglobin concentration; PLT, platelet) (Liu et al., 2020).

To evaluate the extended biosafety of DHPC, Male ICR mice (approximately 20.0 g) were divided into PBS group and DHPC group, with three mice in each group. Mice received intraperitoneal injections of PBS or DHPC at a dose of 0.5 mg/kg every other day for a total of five injections (on days 1, 3, 5, 7, and 9). On day 14, blood samples were collected from the retro‑orbital plexus. Serum biochemistry markers (ALT, AST, ALP, UREA; CREA) and hematological parameters (WBC, RBC, HGB, HCT, MCV, PLT) were measured (Liu et al., 2025).

## 1.7 Enzyme-linked immunosorbent assay (ELISA)

Commercial ELISA kits (EM0183, EM0121; Fine Biotech, China) were used to quantify TNF-α and IL-6 levels in wound tissue samples, as per the manufacturer's protocols.

## 1.8 Hemolysis test

Fresh mouse blood was centrifuged (1500 rpm, 15 min) to pellet red blood cells, which were subjected to three washes with physiological saline and diluted to a 5% suspension. The suspension was incubated with water, PBS, or DHPC (10, 25, 50 µM) for 3 h at 37°C. The supernatant absorbance at 540 nm was recorded following centrifugation at 11,000 rpm for 5 min, and the hemolysis rate was expressed as a percentage of that observed in the water-treated group (Ullah et al., 2024).

# 2 Supplementary Figures

**Figure S1.** ^1^H NMR spectrum of DHPC.

**Figure S2.** ^13^C NMR spectrum of DHPC.

**Figure S3.** Mass spectrum of DHPC.

**Figure S4.** Detection of singlet oxygen, superoxide anion, and hydroxyl radical generated by DHPC (8 μM) after light irradiation (637 nm; 20 mW/cm^2^). Data are presented as mean ± SD (n = 3). *****P* < 0.0001 (two-way ANOVA, Tukey’s *post-hoc* test).

**Figure S5.** UV-vis spectra of DHPC (10 μM) in DMSO (A) and PBS (B) after light irradiation.

**Figure S6.** Survival percentage of MRSA and *P. aeruginosa* after treatment with DHPC (12 μM), with or without washing, followed by light irradiation (20 mW/cm^2^, 10 min). Survival was expressed as percentage of PBS+D controls. Data are presented as mean ± SD (n = 3). *****P* < 0.0001 (two-way ANOVA, Dunnett’s *post-hoc* test). D, dark; L, light.

**Figure S7.** Survival percentage of MRSA and *P. aeruginosa* after treatment with DHPC (12 μM) + light (20 mW/cm^2^, 10 min) or MB (12 μM) + light (20 mW/cm^2^, 10 min). Survival was expressed as percentage of PBS + Dark controls. Data are presented as mean ± SD (n = 3). *****P* < 0.0001 (two-way ANOVA, Tukey’s *post-hoc* test).

**Figure S8.** ATP levels in MRSA and *P. aeruginosa* after treatment with DHPC (12 and 16 μM, respectively) + light (20 mW/cm^2^, 10 min). Data are presented as mean ± SD (n = 5). *****P* < 0.0001, ns, *P* ≥ 0.05 (two-way ANOVA, Dunnett’s *post-hoc* test). D, dark; L, light.

**Figure S9.** Fluorescence imaging of MRSA and *P. aeruginosa* after incubation with DHPC (20 μM, 20 min) followed by washing. The fluorescent signal of DHPC was predominantly associated with the bacterial cell envelope. Scale bar = 5 µm.

**Figure S10.** Cell viability of 4T1 cells treated with DHPC (20 μM) + light (20 mW/cm^2^, 10 min). Data are presented as mean ± SD (n = 3). D, dark; L, light.

**Figure S11.** Survival percentage of MRSA and *P. aeruginosa* following repeated daily exposure to sublethal concentration of DHPC (8 μM and 12 μM respectively) + light (20 mW/cm^2^, 10 min) over 10 consecutive days. Viability was assessed on days 1, 5, and 10. Survival was expressed as percentage of PBS+D controls. Data are presented as mean ± SD (n = 3). *****P* < 0.0001 (two-way ANOVA, Tukey’s *post-hoc* test). D, dark; L, light.

**Figure S12.** Biofilm biomass of MRSA and *P. aeruginosa* after DHPC + light (20 mW/cm^2^, 10 min) treatment followed by 36 h of incubation. Biomass was expressed as percentage of PBS + Dark controls. Data are presented as mean ± SD (n = 3). ****P* < 0.001, *****P* < 0.0001 (two-way ANOVA, Dunnett’s *post-hoc* test).

**Figure S13.** Confocal microscopy images of MRSA and *P. aeruginosa* biofilms (24 hour old) after incubation with fluorescein diacetate (FDA) and DHPC (28 μM) for 20 min followed by washing. DHPC fluorescence was observed within the biofilm matrix, indicating its penetration.

**Figure S14.** Survival percentage of MRSA and *P. aeruginosa* within biofilms after treatment with DHPC (16 μM) + light (20 mW/cm^2^, 10 min) or MB (16 μM) + light (20 mW/cm^2^, 10 min). Survival was expressed as percentage of PBS + Dark controls. Data are presented as mean ± SD (n = 3). *****P* < 0.0001 (two-way ANOVA, Tukey’s *post-hoc* test).

**Figure S15.** Biofilm biomass of mature MRSA and *P. aeruginosa* biofilms (cultured for 36 h) after MB + light (20 mW/cm^2^, 10 min) treatment at concentrations of 24 μM and 28 μM, respectively. Biomass was expressed as percentage of PBS+D controls. Data are presented as mean ± SD (n = 3). ****P* < 0.001, *****P* < 0.0001 (two-way ANOVA, Dunnett’s *post-hoc* test).

**Figure S16.** (**A**) Body weight changes of mice during the 10-day treatment period, measured every two days for 10 d. Data are presented as mean ± SD (n = 6). (**B**) Microscopic images of H&E staining of the major organs (lung, liver, spleen, kidney and heart) from mice subjected to different treatments. Scale bar = 200 μm. (**C**) Hemolysis ratio of mouse blood samples following different treatments. Water-treated samples served as the positive control. Data are presented as mean ± SD (n = 3). D, dark; L, light.

**Figure S17.** Hematological parameters measured in mice on day 10. WBC, white blood cell count; LY, lymphocyte count; RBC, red blood cell count; HGB, hemoglobin; HCT, hematocrit; MCV, mean corpuscular volume; MCH, mean corpuscular hemoglobin; MCHC, mean corpuscular hemoglobin concentration; PLT, platelet count. Data are presented as mean ± SD (n = 3). ns, *P* ≥ 0.05 (two-way ANOVA, Tukey’s *post-hoc* test). L, light.

**Figure S18.** (**A**) Body weight changes of mice measured every two days over 14 days. Data are shown as mean ± SD (n = 3). (**B-F**) Evaluation of serum biochemical markers in mice at 14 d. Data are presented as mean ± SD (n = 3). ns, *P* ≥ 0.05 (two-way ANOVA, Tukey’s *post-hoc* test). (**G-L**) Hematological parameters measured in mice on day 14. Data are presented as mean ± SD (n = 3). ns, *P* ≥ 0.05 (two-way ANOVA, Tukey’s *post-hoc* test). (**M**) Microscopic images of H&E staining of the major organs (heart, liver, spleen, lung, and kidney) from mice at 14 d. Scale bar = 150 μm.

**References**

Li, J., Luo, Y., Zeng, Z., Cui, D., Huang, J., Xu, C., et al. (2022). Precision cancer sono-immunotherapy using deep-tissue activatable semiconducting polymer immunomodulatory nanoparticles. *Nat. Commun.* 13:4032. doi:10.1038/s41467-022-31551-6

Li, W. P., Su, C. H., Wang, S. J., Tsai, F. J., Chang, C. T., Liao, M. C., et al. (2017). CO(2) Delivery To Accelerate Incisional Wound Healing Following Single Irradiation of Near-Infrared Lamp on the Coordinated Colloids. *ACS Nano* 11, 5826-5835. doi:10.1021/acsnano.7b01442

Liu, T., Xiao, B., Xiang, F., Tan, J., Chen, Z., Zhang, X., et al. (2020). Ultrasmall copper-based nanoparticles for reactive oxygen species scavenging and alleviation of inflammation related diseases. *Nat. Commun.* 11:2788. doi:10.1038/s41467-020-16544-7

Liu, Z., Li, M., Xie, Q., Liu, Y., Huang, J., Zeng, Q., et al. (2025). Eradicating fungal biofilm-based infections by ultrasound-assisted semiconductor sensitized upconversion photodynamic therapy. *Nat. Commun.* 16:6499. doi: 10.1038/s41467-025-61519-1

Qi, S., Kwon, N., Yim, Y., Nguyen, V. N., and Yoon, J. (2020). Fine-tuning the electronic structure of heavy-atom-free BODIPY photosensitizers for fluorescence imaging and mitochondria-targeted photodynamic therapy. *Chem. Sci.* 11, 6479-6484. doi:10.1039/d0sc01171a

Su, Z., Xu, D., Hu, X., Zhu, W., Kong, L., Qian, Z., et al. (2024). Biodegradable oxygen-evolving metalloantibiotics for spatiotemporal sono-metalloimmunotherapy against orthopaedic biofilm infections. *Nat. Commun.* 15:8058. doi:10.1038/s41467-024-52489-x

Ullah, I., Khan, S. S., Ahmad, W., Liu, L., Rady, A., Aldahmash, B., et al. (2024). NIR light-activated nanocomposites combat biofilm formation and enhance antibacterial efficacy for improved wound healing. *Commun. Chem.* 7:131. doi:10.1038/s42004-024-01215-1

Zhu, L., Song, G., Zhang, W., Wu, Y., Chen, Y., Song, J., et al. (2025). Aggregation induced emission luminogen bacteria hybrid bionic robot for multimodal phototheranostics and immunotherapy. *Nat. Commun.* 16:2578. doi:10.1038/s41467-025-57533-y
